# Q fever seroprevalence in parturient women: the EQRUN cross-sectional study on Reunion Island

**DOI:** 10.1186/s12879-020-04969-w

**Published:** 2020-04-03

**Authors:** Julien Jaubert, Laura Atiana, Sophie Larrieu, Philippe De Vos, Claudine Somon-Payet, Sylvaine Porcherat, Yoan Mboussou, Florence Naze, Sandrine Picot, Malik Boukerrou, Pierre-Yves Robillard, Patrick Gérardin

**Affiliations:** 1 Laboratoire de Bactériologie, Virologie et Parasitologie, Centre Hospitalier Universitaire (CHU) de la Réunion, Saint Pierre, Reunion France; 2 CIRE Océan Indien, Santé Publique France, French National Public Health Agency, Saint Denis, Reunion France; 3 Maternité, Clinique Durieux, Le Tampon, Reunion France; 4 Maternité, Pôle Femme Mère Enfant, CHU de la Réunion, St Pierre, Reunion France; 5 INSERM CIC 1410 Epidémiologie Clinique, Centre Hospitalier Universitaire, Groupe Hospitalier Sud Réunion, CHU Réunion, BP 350, 97448 Saint Pierre, Cedex-Reunion France; 6 CEPOI-EA7388, Pôle Femme Mère Enfant, CHU de la Réunion, Saint Pierre, Reunion France; 7 UM 134 PIMIT Processus Infectieux en Milieu Insulaire Tropical, Université de La Réunion, INSERM 1187, CNRS 9192, IRD 249, CYROI, Sainte Clotilde, Reunion France

**Keywords:** Immunofluorescence, Cross sectional study, *Q fever*, *Coxiella burnetii*, Pregnancy, Childbirth, Parturient woman, Prevalence, Prevalence proportion ratio, Propensity score matching

## Abstract

**Background:**

Q fever (*Coxiella burnetii* infection) has been associated with adverse perinatal outcomes. After investigating the obstetrical importance of Q fever on Reunion island and demonstrating an association between incident Q fever and miscarriage, we conducted a cross-sectional serosurvey to assess the prevalence of *Coxiella burnetii* infection among parturient women.

**Methods:**

Between January 9 and July 24, 2014, within the level-4 maternity of Saint Pierre hospital and the level-1 maternity of Le Tampon, we proposed to screen all parturient women for *Coxiella burnetii* serology. Seropositivity was defined using indirect immunofluorescence for a dilution of phase 2 IgG titre ≥1:64. Further dilutions were chosen to discriminate recent or active infections from past or prevalent infections (< 1:128) and classify these as either possible (1:128), or probable (≥1:256). Recurrent miscarriage, stillbirth, preterm birth, small-for-gestational as well as a composite outcome of these adverse pregnancy outcomes were compared according to seropositivity using bivariate analysis or propensity score matching of seropositive and seronegative women on confounding factors.

**Results:**

Among 1112 parturient women screened for Q fever over this 7-month period, 203 (18.3%) were seropositive. Overall weighted seroprevalence was of 20.1% (95%CI, 17.7–22.5%). Weighted seroprevalence of probable infections was 4.7% (95%CI 3.4–5.9%), while > 90% of positive serologies corresponded to past infections or false positives. Seropositivity was associated with none of the abovementioned adverse perinatal outcomes, whether in unpaired or matched analyses on propensity score.

**Conclusion:**

The magnitude and the pattern of seroprevalence suggest that Q fever is endemic on Reunion island. In this context, we found no significant contribution of prevalent *Coxiella burnetii* infection to adverse pregnancy outcomes. Although reassuring, these data put in our endemic context, with a previously demonstrated increased risk of incident Q fever associated miscarriage, encourage us to protect pregnant women against the risk of new infection, periconceptional or early in pregnancy.

## Background

*Coxiella burnetii* infection, best known as Q fever in humans, is a zoonotic disease, which has been reported from almost every country worldwide [1]. *Coxiella burnetii* is an obligate Gram negative intracellular bacterium that resides in wild and domesticated animals. Cattle, goats and sheep serve as reservoir to spread the bacterium to human populations [1, 2]. This pathogen exhibits a strong tropism for the reproductive apparatus, which is the cause of complications, including spontaneous abortion (miscarriage), preterm delivery and foetal deaths [2]. Humans may be infected directly through handling of birth products or contact with bodily fluids, but most of the disease burden is believed to come from infected aerosols of farm animals [1].

In the sero-epidemiologic studies of pregnant woman, Q fever has been associated inconsistently with miscarriage [3–5], preterm birth [6–8], or low birthweight [8], and infrequently with foetal death [9], or congenital malformations [9], whilst small-for-gestational age (intrauterine growth restriction) and oligohydramnios are classical complications only reported from case series [10–12]. These adverse pregnancy outcomes (APOs) have been associated with both acute and persistent Q fever infections [1]. They are likely the consequence of a placental immune dysregulation with an interleukin-10 overproduction, subsequent silencing of the dendritic cells, which favours bacterial replication within the trophoblast cell vacuoles of the allantochorion that express lysosomal markers [1]. Notwithstanding, causal relationship between a positive *Coxiella burnetii* serology and APOs remains elusive given discrepancies between case series and observational studies.

Following the documentation of Q fever endocarditis on Reunion island [13], demonstration of a significant contribution of acute infections to miscarriage and, to lesser extent, stillbirth [14], we conducted a cross-sectional serosurvey to assess the prevalence of *Coxiella burnetii* antibodies among parturient women. Our secondary objectives were to search for risk factors and evaluate the contribution of seropositivity to APOs.

## Methods

### Setting and population

The characteristics of the study place have been described previously [14] and can be found at https://bmcinfectdis.biomedcentral.com/articles/10.1186/s12879-019-4619-6.

Between January 9 and July 24, 2014, all parturient women attending the regional perinatal healthcare centre of Saint Pierre hospital (level-4 maternity) and the private maternity of Le Tampon (level-1 maternity) were asked to be screened for *Coxiella burnetii* serology in addition to the usual data collection of a birth registry [15, 16].

#### Serology

Sera were tested using an indirect fluorescent antibody (IFA) assay with commercially available antigens for *Coxiella burnetii* (*Coxiella burnetti* I + II IFA IgG/IgM/IgAt®, Vircell, Grenade, Spain). Seropositivity was defined for a phase 2 or phase 1 IgG titre ≥1:64 with or without phase 2/1 IgM ≥ 1:48. Further dilutions were chosen to discriminate recent or active infections from past or prevalent infections (IgG2 < 1:128 and IgM2 < 1:48) and classify these as either possible (IgG2 = 1:128 and/or IgM2 ≥ 1:48), or probable (IgG2 ≥ 1:256 whatever IgM level). The use of phase 2 IgM alone were deemed only suggestive of recent infection and did not enter in the case definitions, as recommended by Netherlands experts [17]. These thresholds were chosen conservative to fulfil the National Reference Centre requirements and minimize the false positives [18]. Persistent infection was defined for a phase 1 to phase 2 IgG ratio > 1 in the absence of IgM antibodies [19].

#### Statistical analysis

Statistical analyses were performed using Stata 14.2® (StataCorp, College Station; Texas, USA). Crude seroprevalence rates were estimated with 95% confidence intervals (CI), next they were weighted on the maternity of childbirth, marital status, country of birth, education, occupation and a homemade social deprivation index [20] to account for the structure of the reproductive population.

Associations between maternal variables (maternity of childbirth, residence area, neighbourhood deprivation, age, origin, marital status, education, occupation and parity) and seropositive status were determined using crude and weighted chi-square tests, unadjusted and population-readjusted log-binomial models to identify potential risk factors. In these, prevalence proportion ratios (PPR) and 95%CI were estimated as association measures.

Recurrent miscarriage (foetal demise < 22 weeks or ≤ 500 g.), stillbirth (foetal death ≥22 weeks or > 500 g.), preterm birth (< 37 weeks), small-for-gestational age (birthweight <10th percentile), congenital malformations (ICD-10 codes), oligohydramnios or polyhydramnios, as well as a composite outcome of these APOs were compared according to *Coxiella burnetii* antibodies using bivariate analysis or propensity score matching of seropositive and seronegative women on putative confounders with complete data, namely maternal hypertension, diabetes, addiction and foetal gender. All these estimations were re-adjusted using sampling fractions to account for selection bias. A *P* value < 0.05 was considered significant.

## Results

**Fig. 1:**
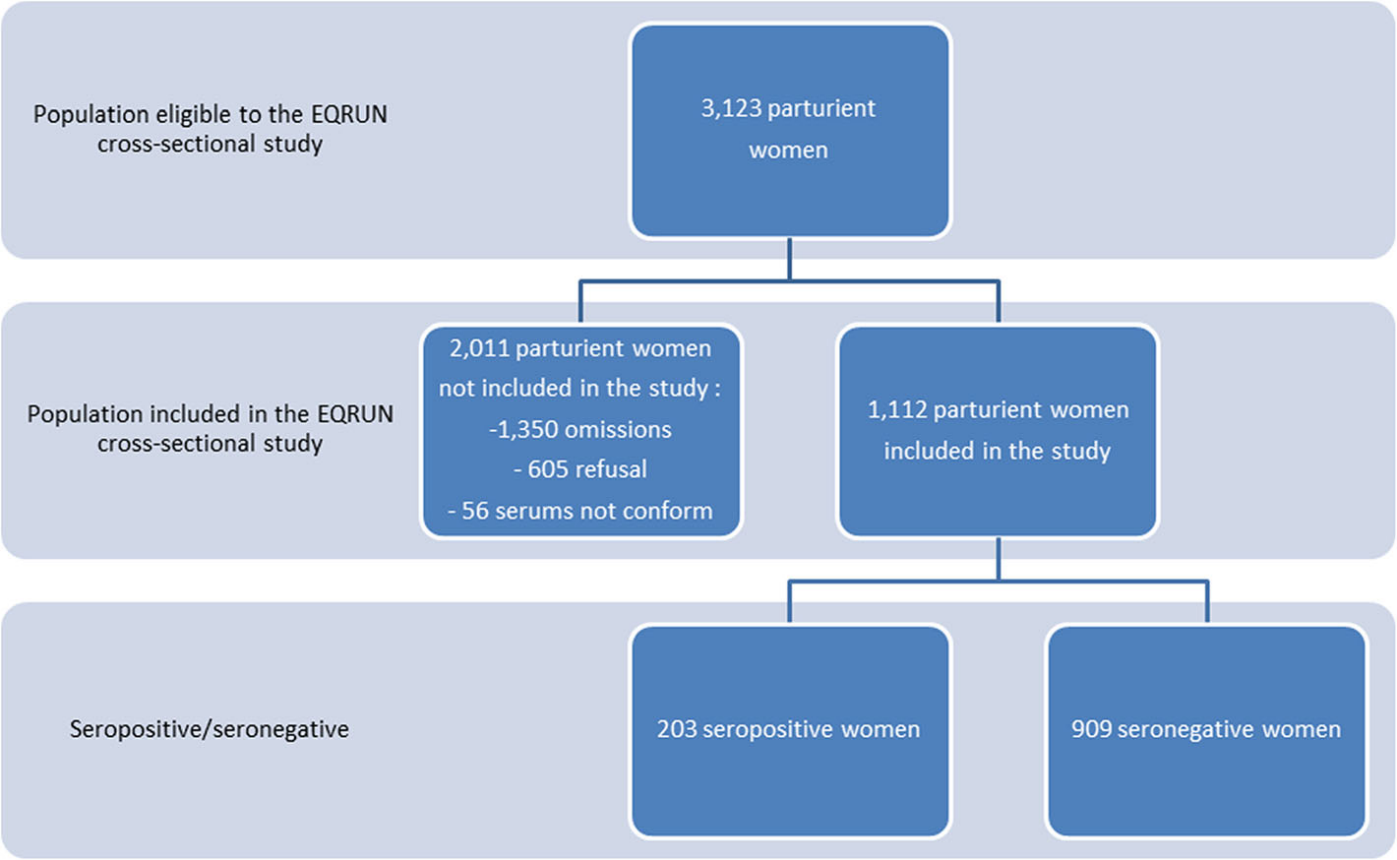
Flow chart of the study population

Over a 7-month period, 3123 pregnant women delivered in the southern Reunion island maternities. Among these, 1112 parturient women were screened for Q fever (Fig. 1). The participation was higher in the level-1 maternity than in the level-3 maternity (80% versus 25%). The sample studied differed from the reproductive population for several maternal characteristics including the maternity of childbirth, neighbourhood deprivation, marital status, education, and occupation (Table S1), which imposed to weight the analysis on these variables to control the selection bias.

Seropositivity rate of Q fever was of 18.3% (203/1112) and weighted seropositivity rate was of 20.1% (95%CI, 17.7–22.5%), among which a range of 93.4 to 96.1% corresponded to past infections or false positives (Phase 2 IgM < 1:48). These figures were respectively of 12.3% (137/1112) and 14.1% (95%CI, 12.0–16.1%) with the more stringent cut-off ≥1:128 suggestive of possible infections, among which a range of 5.9 to 9.0% corresponded to recent or active infections (Phase 2 IgM ≥ 1:48). At dilutions ≥1:256 indicative of probable infections, seroprevalence and weighted seroprevalence were of 4.0% (45/11112) and 4.7% (95%CI, 3.4–5.9%), respectively, which gave potential to six recent or active infections of putative gestational onset (Phase 2 IgM ≥ 1:48). Of three women harbouring phase 1 IgG antibodies, one met the definition of a persistent infection with a titre of 1:128. The detail of the serologic responses to *Coxiella burnetii* antigens is displayed in Table 1.

**Table 1:** Serologic responses to phase 2 IgM (recent infection), phase 1 IgG (persistent infection) and phase 2 IgG (recent/active infection) antigens among 1112 parturient women in southern Reunion island, January to July 2014

Peripheral blood titre	Phase 2 IgM n (%)	Phase 1 IgG n (%)	Phase 2 IgG n (%)	Phase 2 IgM + IgG, n (%)	Phase 2 + 1 IgG, n (%)
**1:48**	15 (1.3)	–	–	15 (1.3)	–
**1:64**	–	3 (0.3)	203 (18.3)	15 (1.3)	3 (0.3)
**1:96**	12 (1.1)	–	–	15 (1.3)	–
**1:128**	–	1 (0.1)	137 (12.3)	15 (1.3)	1 (0.1)
**1:192**	9 (0.8)	–	–	9 (0.8)	–
**1:256**	–	0 (0.0)	45 (4.0)	6 (0.5)	0 (0.0)
**1:384**	3 (0.3)	–	–	2 (0.2)	–
**1:512**	–	0 (0.0)	22 (1.9)	–	0 (0.0)

Data are decremental as the dilution progresses

None of the abovementioned eight maternal characteristics were associated with the seropositive status in bivariate analysis (Table 2). Single women, middle school-educated women, nulliparous or multiparous women were more likely to be seropositive in population-readjusted analysis (Table S2). Importantly, neither the maternal occupation nor the location of the residence area was associated with seropositivity in both types of analysis. Further dilutions failed to identify risk factors.

**Table 2:** Maternal characteristics associated with Q fever seropositivity in bivariate analysis, among 1112 parturient women, Reunion island, January to July 2014

Outcome: ***Coxiella burnetii*** Phase 2 IgG ≥ 1:64					
Exposure variables	n	Crude %	Crude PPR	95% CI	***P value***
**Maternity centre**					0.146
Level-4, Saint Pierre	127 / 645	19.7	1.21	0.93–1.57	
Level-1, Le Tampon	76 / 467	16.3	1		
**Area of residence**					0.563
North or East	1 / 4	25.0	1		
West	12 / 55	21.8	0.87	0.14–5.12	
South	174 / 939	18.5	0.74	0.13–4.07	
**Neighbourhood deprivation**^**a**^					0.838
Minimum	91 / 477	19.1	1		
Intermediate	66 / 346	19.1	1.00	0.75–1.33	
High	30 / 175	17.1	0.89	0.61–1.31	
**Age**					0.172
≤ 25 years	81 / 424	19.1	1		
26–31 years	50 / 333	15.0	0.79	0.57–1.08	
32–47 years	72 / 355	20.3	1.06	0.80–1.41	
**Place of birth**					0.302
Reunion	141 / 771	18.3	1.08	0.74–1.57	
Indian ocean	17 / 67	25.4	1.50	0.88–2.57	
Metropolitan France	27 / 160	16.9	1		
**Marital Status**					0.076
In couple	125 / 723	17.3	1		
Celibacy	60 / 270	22.2	1.29	0.97–1.69	
**Education**					0.349
Primary school	9 / 44	20.5	1.24	0.65–2.33	
Middle school	38 / 165	23.0	1.39	0.96–2.02	
High school	77 / 383	20.1	1.21	0.88–1.67	
University	54 / 327	16.5	1		
**Occupation**					0.421
Unemployed	120 / 601	20.0	1.19	0.90–1.56	
Farmer	0 / 2	0.0	NA		
Other work	67 / 400	16.7	1		
**Parity**					0.236
Nullipara	77 / 407	18.9	1.17	0.85–1.60	
Primipara	55 / 340	16.2	1		
Multipara	57 / 264	21.6	1.33	0.95–1.86	

Data are numbers, crude seropositive rates (%), crude prevalence proportion ratios (PPR) and 95% confidence intervals (95% CI). NA: not assessed. *P* values are given for Pearson chi2 tests and not for Wald tests

^a^Derived from a homemade social deprivation index categorising the 24 municipalities of the island into tree levels based on three indices: socio-economic composition (three variables) [20]

Seropositivity was not associated with any of the abovementioned APOs, whether in unpaired bivariate (Table 3), population-readjusted, or matched analyses (Table S3). Further adjustments on pregnancy related hypertensive disorders, diabetes, maternal addictions (smoking or alcohol), multiple pregnancy, or foetal gender, did not increase the risks of APOs with Q fever. Among the outcomes of the six women with recent or active infections of putative gestational onset, we noticed one small-for-gestational neonate, and one oligohydramnios. Of note, the outcome of phase 1 IgG was without harm to the foetus.

**Table 3:** Adverse pregnancy outcomes associated with Q fever seropositivity in bivariate analysis, among 1112 parturient women, Reunion island, January to July 2014

Adverse	**Exposure variable:*Coxiella burnetii*Phase 2 IgG ≥ 1:64**				
pregnancy outcomes	n	Crude %	Crude PPR	95% CI	***P value***
**Composite outcome**^**a**^					0.567
In seropositive	52 / 203	25.6	0.93	0.71–1.20	
In seronegative	251 / 909	27.5	1		
**Preterm birth**					0.775
In seropositive	11 / 203	5.4	0.91	0.48–1.71	
In seronegative	54 / 909	5.9	1		
**Small-for-gestational age**					0.823
In seropositive	41 / 203	20.2	0.97	0.71–1.31	
In seronegative	190 / 909	20.9	1		
**Exposure variable**: ***Coxiella burnetii*Phase 2 IgG ≥ 1:256 or Phase 2 IgM ≥ 1:48**					
**Composite outcome**^**a**^					0.801
In seropositive	13 / 45	28.9	1.06	0.66–1.70	
In seronegative	290 / 1067	27.2	1		
**Preterm birth**					0.319
In seropositive	1 / 45	2.2	0.37	0.05–2.61	
In seronegative	64 / 1067	6.0	1		
**Small-for-gestational age**					0.897
In seropositive	9 / 45	20.0	0.96	0.53–1.74	
In seronegative	222 / 1067	20.8	1		

Data are numbers, crude seropositive rates (%), crude prevalence proportion ratios (PPR) and 95% confidence intervals (95% CI). NA: not assessed. *P* values are given for Wald tests and not for crude Pearson chi2 tests

^a^Recurrent miscarriage, stillbirth, or preterm birth, small-for-gestational age, congenital malformations, oligohydramnios or polyhydramnios

During the study period, 143 serologies were performed to diagnose Q fever outside the maternity, in various clinical indications. Among these, clinical and microbiological evaluation identified nine recent or active infections (phase 2 IgG ≥ 1:256 and phase 2 IgM: ≥1:48) and six possible recent or active infections (phase 2 IgG 1:128 and phase 2 IgM: ≥1:48), which leads to a community-level annual incidence rate of 25.7 per 100,000 inhabitants.

## Discussion

*Q* fever is widely distributed in tropical areas and considered as endemic in Africa [1]. In the neighbouring Indian ocean, first isolations of *Coxiella burnetii* go back from the 1950’s and sporadic infections have been reported both in autochthonous and traveller populations returning from Comoros, Madagascar or Reunion island [13]. In La Réunion, the overall seropositivity and shedding rates were of 11.8 and 0.8% in cattle, 1.4 and 4.4% in sheep and 13.4 and 20.1% in goats, respectively [2]. In humans, a population-based serosurvey conducted on stored frozen samples dated 2009 estimated the exposure around 6% [21].

Herein, we confirm the exposure of Reunion island pregnant women to autochthonous transmission of *Coxiella burnetii*. In agreement, we evidenced a threefold higher seropositivity rate in parturient women than in the general population, but similar seroprevalences, around 4%, with respect to probable infections. The discrepancy between the two populations in seropositivity rates could stem from a recruitment bias, our study having been conducted exclusively in the South Reunion island maternities that welcome the Southern and Western pregnant populations, the more exposed to small-ruminant farms and tradewinds [2]. Cross reactions are unlikely, given their proportion was slight (4%) in the general population [21]. The congruence in the seroprevalence of acute infections may advocate equality of the two populations against the risk of an unrecognized airborne threat.

Compared to other seroepidemiologic studies found in the literature, the seropositivity rate found in Reunionese pregnant women was 2 to 5-fold higher than those observed in endemic western countries [6, 8, 22] and on average, far higher than those retrieved from Caribbean islands [23], while the seroprevalence of acute infections was 20 to 30-fold higher than those observed in endemic [7, 8, 24] or hyperendemic settings (Table S4) [25]. Together with a high magnitude incidence, these sero-epidemioologic data may account for an endemic setting, or even an unrecognized hyperendemic setting.

Given institutional constraints, we were unable to deploy the initial investigation that had been planned to include both individual and contextual risk factors, so that we did have access only to routine birth registry data. Thereby, we failed to identify risk factors from our serosurvey. Importantly, it was shown both in the Netherlands and Denmark that pregnant women living in the vicinity of livestock animals [24], especially in the vicinity of goat farms [22], exhibit higher phase 2 antibody titres and were more likely to develop an acute infection than women not exposed to small ruminants.

Of note, seropositivity was not associated with adverse pregnancy outcomes (*i.e*, that means that seropositivity was more likely reminiscent of past infection than of recent or active infection during the pregnancy), whether in unpaired or matched analysis, which strongly contrasts with incident (i.e.*,* active) infections that were responsible of miscarriages, and to a lesser extent, of stillbirths in a preliminary prospective cohort study [14]. This is consistent with previous knowledge on Q fever that is well known to be abortive [1, 2, 4] and to cause intrauterine foetal death [9]. This is also coherent with data acquired from TORCH pathogens [26] or congenital flavivirus infections [27, 28], e.g., zika virus or dengue virus, for which the active or symptomatic character of the infection increases the risk of vertical transmission and adverse pregnancy outcomes. Importantly, none of the women harbouring phase 1 IgG was symptomatic or delivered an infant with an adverse foetal outcome, which does no plaid for the use of a low phase 1 IgG cut-off or the phase 1 to phase 2 IgG ratio alone to define persistent infection.

### Limitations

#### Limitations of the study

This study has potential limitations. First, it may be prone to residual selection bias given participation rate was much lower than anticipated, and the study sample was skewed towards women living in better socio-economic conditions (more advantageous neighbourhoods, birthplace in mainland France, living in couple, higher level of education). This may persist even despite reweighting our sample to compensate this limitation. Second, we cannot exclude a misclassification bias owed to serology cross reactions between *Coxiella burnetii* and *Rickettsia* species, given the high proportion (33%) of cross-reactive sera observed in the community [21]. However, given people with cross-reactive serum were at 70% located in the western dryer part of the island area also exposed to murine typhus [29], we believe that the pregnant women sampled in our study, mostly based in South Reunion, were less likely prone to multiple infections than women dwelling in the West, so this was unlikely to change the overall magnitude of the reproductive population exposure to *Coxiella burnetii*. These things being said, we concede the need of more specific methods for interpreting serosurvey data, such as Western Blot or seroneutralization, allowing the identification at the species level.

## Conclusions

The magnitude and the pattern of seroprevalence in pregnant women suggest that Q fever is endemic on Reunion island. In this context, we found no significant contribution of prevalent *Coxiella burnetii* infections in adverse pregnancy outcomes. These results are reassuring for the population of childbearing age and contrast with those of incident *Coxiella burnetii* infection that are associated with an increased risk of miscarriage, or even with an increased risk of stillbirth. In accordance, we advocate as mitigation measure aimed at limiting the burden of Q fever on reproduction that pregnant women should be kept away from farms to rule out airborne transmission, avoid direct contact with ruminants or their byproducts, or to consume uncooked farm products.

## Supplementary information

**Additional file 1: Table S1.** Data are case numbers (N and n) and percentages. #Derived from a homemade social deprivation index categorising the 24 municipalities of the island into tree levels based on three indices: socio-economic composition (three variables) [20]. **Table S2.** Data are numbers, weighted seropositive rates (%), population-readjusted prevalence proportion ratios (PPR) and 95% confidence intervals (95% CI). NA: not assessed. P values are given for weighted chi2 tests and not for Wald tests. #Derived from a homemade social deprivation index categorising the 24 municipalities of the island into tree levels based on three indices: socio-economic composition (three variables) [20]. **Table S3.** Data are numbers that are not cumulative, crude seropositive rates (%), population-readjusted prevalence proportion ratios (PPR), matched odd ratios (OR), and 95% confidence intervals (95% CI). NA: not assessed. P values are given for Wald tests and not for Pearson chi2 tests. *Recurrent miscarriage, stillbirth, or preterm birth, small-for-gestational age, congenital malformations, oligohydramnios or polyhydramnios. Seropositive women were matched with as many seronegative women as possible on maternal hypertension, diabetes, addiction and foetal gender. **Table S4.** Data are percentages.

## Data Availability

The dataset generated and/or analysed during the current study are not publicly available due to anonymity policy issues but are available from the corresponding author on request.

## Abbreviations

APOs: Adverse pregnancy outcomes
CPP: Comité de Protection des Personnes
IFA: Indirect fluorescent antibody (alternatively taken as immunofluorescent assay)
IgA: Immunoglobulin A
IgG: Immunoglobulin G
IgG2: Phase 2 immunoglobulin G
IgM: Immunoglobulin M
95%CI: 95% confidence interval
PPR: Prevalence proportion ratio
TORCH: Toxoplasmosis, Other (syphilis, varicella-zoster, parvovirus B19), Rubella, Cytomegalovirus, Herpes)
