# Supplementary material for: Q fever seroprevalence in parturient women: the EQRUN cross-sectional study on Reunion Island
Source: BMC Infect Dis. 2020 Apr 3;20:261. doi: 10.1186/s12879-020-04969-w (PMC7118902; doi:10.1186/s12879-020-04969-w)
Supplement: Supplementary file 1 — Additional file 1: Table S1. Data are case numbers (N and n) and percentages. #Derived from a homemade social deprivation index categorising the 24 municipalities of the island into tree levels based on three indices: socio-economic composition (three variables) [20]. Table S2. Data are numbers, weighted seropositive rates (%), population-readjusted prevalence proportion ratios (PPR) and 95% confidence intervals (95% CI). NA: not assessed. P values are given for weighted chi2 tests and not for Wald tests. #Derived from a homemade social deprivation index categorising the 24 municipalities of the island into tree levels based on three indices: socio-economic composition (three variables) [20]. Table S3. Data are numbers that are not cumulative, crude seropositive rates (%), population-readjusted prevalence proportion ratios (PPR), matched odd ratios (OR), and 95% confidence intervals (95% CI). NA: not assessed. P values are given for Wald tests and not for Pearson chi2 tests. *Recurrent miscarriage, stillbirth, or preterm birth, small-for-gestational age, congenital malformations, oligohydramnios or polyhydramnios. Seropositive women were matched with as many seronegative women as possible on maternal hypertension, diabetes, addiction and foetal gender. Table S4. Data are percentages. [file 12879_2020_4969_MOESM1_ESM.docx]

| **Table S1. Maternal characteristics of the South Réunion island reproductive population and of the E-Q-RUN study population, January to July 2014** | | | | | |
| --- | --- | --- | --- | --- | --- |
|  | **Reproductive population** | | **Study population ^†^** | |  |
| **Variables** | **N** | **%** | **n** | **%** | ***P value*** |
| **Maternity centre** |  |  |  |  | < 0.001 |
| Level-4, Saint Pierre | 1,893 | 94.1 | 645 | 58.0 |  |
| Level-1, Le Tampon | 118 | 5.9 | 467 | 42.0 |  |
| **Area of residence** |  |  |  |  | 0.642 |
| North or East | 12 | 0.6 | 4 | 0.4 |  |
| West | 109 | 5.9 | 55 | 5.5 |  |
| South | 1,732 | 93.5 | 939 | 94.1 |  |
| **Neighbourhood deprivation^#^** |  |  |  |  | < 0.001 |
| Minimum | 672 | 36.3 | 477 | 47.8 |  |
| Intermediate | 699 | 37.7 | 346 | 34.7 |  |
| High | 482 | 26.0 | 175 | 17.5 |  |
| **Age** |  |  |  |  | 0.190 |
| ≤ 25 years | 653 | 34.9 | 354 | 31.8 |  |
| 26-31 years | 665 | 35.5 | 403 | 36.2 |  |
| 32-47 years | 553 | 29.6 | 355 | 31.9 |  |
| **Place of birth** |  |  |  |  | 0.002 |
| Reunion | 1,452 | 78.5 | 771 | 77.3 |  |
| Indian ocean | 171 | 9.3 | 67 | 6.7 |  |
| Metropolitan France | 222 | 12.2 | 160 | 16.0 |  |
| **Marital Status** |  |  |  |  | < 0.001 |
| In couple | 1,177 | 63.7 | 723 | 72.8 |  |
| Celibacy | 672 | 36.3 | 270 | 27.2 |  |
| **Education** |  |  |  |  | < 0.001 |
| Primary school | 66 | 3.7 | 44 | 4.8 |  |
| Middle school | 465 | 26.1 | 165 | 17.9 |  |
| High school | 747 | 42.0 | 383 | 41.7 |  |
| University | 502 | 28.2 | 327 | 35.6 |  |
| **Occupation** |  |  |  |  | < 0.001 |
| Unemployed | 1,244 | 67.4 | 601 | 59.9 |  |
| Farmer | 5 | 0.3 | 2 | 0.2 |  |
| Other work | 598 | 32.4 | 400 | 39.9 |  |
| **Parity** |  |  |  |  | 0.065 |
| Nullipara | 742 | 39.9 | 407 | 40.3 |  |
| Primipara | 563 | 30.3 | 340 | 33.6 |  |
| Multipara | 554 | 29.8 | 264 | 26.1 |  |
| Data are case numbers (N and n) and percentages. **^#^**Derived from a homemade social deprivation index categorising the 24 municipalities of the island into tree levels based on three indices: socio-economic composition (three variables) [20]. | | | | | |

| **Table S2. Maternal characteristics associated with Q fever seropositivity in population-readjusted analysis, among 1,112 parturient women, Reunion island, January to July 2014** | | | | | | | |
| --- | --- | --- | --- | --- | --- | --- | --- |
| **Outcome: *Coxiella burnetii* IgG ≥ 1:64** | | | | | | | |
| **Exposure variables** | **N** | **Weighted**  **%** | **Population-readjusted PPR** | **95% CI** | ***P value*** | | |
| **Maternity centre** |  |  |  |  | 0.210 | | |
| Level-4, Saint Pierre | 127/645 | 20.7 | 1.25 | 0.86 - 1.82 |  | | |
| Level-1, Le Tampon | 76/467 | 16.5 | 1 |  |  | | |
| **Area of residence** |  |  |  |  | 0.376 | | |
| North or East | 1 / 4 | 9.2 | 1 |  |  | | |
| West | 12 / 55 | 29.3 | 3.19 | 0.23 - 42.94 |  | | |
| South | 174 / 939 | 20.1 | 2.19 | 0.15 - 32.00 |  | | |
| **Neighbourhood deprivation^#^** | |  |  |  | 0.792 | | |
| Minimum | 91 / 477 | 20.8 | 1 |  |  | | |
| Intermediate | 66 / 346 | 21.2 | 1.02 | 0.59 - 1.73 |  | | |
| High | 30 / 175 | 19.7 | 0.94 | 0.69 - 1.29 |  | | |
| **Age** |  |  |  |  | 0.084 | | |
| ≤ 25 years | 81 / 424 | 21.8 | 1 |  |  | | |
| 26-31 years | 50 / 333 | 15.8 | 0.72 | 0.61 - 0.85 |  | | |
| 32-47 years | 72 / 355 | 21.7 | 0.99 | 0.70 - 1.41 |  | | |
| **Place of birth** |  |  |  |  | 0.061 | | |
| Reunion | 141 / 771 | 19.9 | 1.15 | 0.62 - 2.11 |  | | |
| Indian ocean | 17 / 67 | 29.4 | 1.70 | 0.92 - 3.14 |  | | |
| Metropolitan France | 27 / 160 | 17.3 | 1 |  |  | | |
| **Marital Status** |  |  |  |  | < 0.001 | | |
| In couple | 125 / 723 | 18.3 | 1 |  |  | | |
| Celibacy | 60 / 270 | 23.9 | 1.31 | 1.20 - 1.41 |  | | |
| **Education** |  |  |  |  | 0.196 | | |
| Primary school | 9 / 44 | 23.7 | 1.37 | 0.82 - 2.28 |  | | |
| Middle school | 38 / 165 | 23.2 | 1.35 | 1.09 - 1.67 |  | | |
| High school | 77 / 383 | 21.3 | 1.23 | 0.85 - 1.78 |  | | |
| University | 54 / 327 | 17.2 | 1 |  |  | | |
| **Occupation** |  |  |  |  | 0.398 | | |
| Unemployed | 120 / 601 | 21.8 | 1.28 | 0.76 - 2.13 |  | | |
| Farmer | 0 / 2 | 0.0 | NA |  |  | | |
| Other work | 67 / 400 | 17.1 | 1 |  |  | | |
| **Parity** |  |  |  |  | 0.202 | | |
| Nullipara | 77 / 407 | 21.8 | 1.27 | 1.02 - 1.58 |  | | |
| Primipara | 55 / 340 | 17.1 | 1 |  |  | | |
| Multipara | 57 / 264 | 22.2 | 1.30 | 1.02 - 1.64 |  | | |
| Data are numbers, weighted seropositive rates (%), population-readjusted prevalence proportion ratios (PPR) and 95%confidence intervals (95% CI). NA: not assessed. *P* values are given for weighted chi2 tests and not for Wald tests. **^#^**Derived from a homemade social deprivation index categorising the 24 municipalities of the island into tree levels based on three indices: socio-economic composition (three variables) [20]. | | | | | |  |  |

| **Table S3. Adverse pregnancy outcomes associated with Q fever seropositivity in population-readjusted weighted analysis (left side) and propensity score matching analysis (right side), among 1,112 parturient women, Reunion island, January to July 2014** | | | | | | | | |
| --- | --- | --- | --- | --- | --- | --- | --- | --- |
| **Adverse** | **Exposure variable : *Coxiella burnetii* Phase 2 IgG ≥ 1:64** | | | | | | | |
| **pregnancy outcomes** | **n** | **Weighted**  **%** | **Population-readjusted PPR** | **95% CI** | ***P value*** | **Matched**  **OR** | **95% CI** | ***P value*** |
| **Composite outcome*** |  |  |  |  | 0.413 |  |  | 0.471 |
| In seropositive | 52 / 203 | 31.9 | 1.08 | 0.87 - 1.34 |  | 0.87 | 0.87 - 1.34 |  |
| In seronegative | 251 / 909 | 29.5 | 1 |  |  | 1 |  |  |
| **Preterm birth** |  |  |  |  | 0.389 |  |  | 0.621 |
| In seropositive | 11 / 203 | 6.2 | 0.83 | 0.52 - 1.33 |  | 0.83 | 0.39 - 1.74 |  |
| In seronegative | 54 / 909 | 7.5 | 1 |  |  | 1 |  |  |
| **Small-for-gestational age** | |  |  |  | 0.057 |  |  | 0.564 |
| In seropositive | 41 / 203 | 26.4 | 1.23 | 0.99 - 1.51 |  | 0.88 | 0.57 - 1.35 |  |
| In seronegative | 190 / 909 | 21.5 | 1 |  |  | 1 |  |  |
|  | **Exposure variable : *Coxiella burnetii* Phase 2 IgG ≥ 1:256 or Phase 2 IgM ≥ 1:48** | | | | | | | |
| **Composite outcome*** |  |  |  |  | 0.351 |  |  | 0.804 |
| In seropositive | 13 / 45 | 34.3 | 1.15 | 0.82 - 1.60 |  | 1.10 | 0.52 - 2.30 |  |
| In seronegative | 290 / 1,067 | 29.8 | 1 |  |  | 1 |  |  |
| **Preterm birth** |  |  |  |  | 0.315 |  |  | 0.323 |
| In seropositive | 1 / 45 | 3.3 | 0.44 | 0.07 - 2.57 |  | 0.35 | 0.04 - 2.79 |  |
| In seronegative | 64 / 1,067 | 7.4 | 1 |  |  | 1 |  |  |
| **Small-for-gestational age** | |  |  |  | 0.355 |  |  | 0.965 |
| In seropositive | 9 / 45 | 26.5 | 1.19 | 0.79 - 1.77 |  | 1.02 | 0.43 - 2.38 |  |
| In seronegative | 222 / 1,067 | 22.2 | 1 |  |  | 1 |  |  |
| Data are numbers that are not cumulative, crude seropositive rates (%), population-readjusted prevalence proportion ratios (PPR), matched odd ratios (OR), and 95% confidence intervals (95% CI). NA: not assessed. *P* values are given for Wald tests and not for Pearson chi2 tests. *Recurrent miscarriage, stillbirth, or preterm birth, small-for-gestational age, congenital malformations, oligohydramnios or polyhydramnios. Seropositive women were matched with as many seronegative women as possible on maternal hypertension, diabetes, addiction and foetal gender. | | | | | | | | |

| **Table S4. Seropositivity or seroprevalence rates against phase 2 IgM (recent infection), phase 1 IgG (persistent infection) and phase 2 IgG (recent/active infection) in seroepidemiologic studies of pregnant women in the literature and Reunion island (pregnant women, community)** | | | | | | | | | |
| --- | --- | --- | --- | --- | --- | --- | --- | --- | --- |
| **Antibodies**  **Studies, Year ^reference^** | **Phase 2 IgM**  **% (95 % CI)** | | | | **Phase 1 IgG**  **% (95 % CI)** | **Phase 2 IgG**  **% (95 % CI)** | **Phase 2 IgM + IgG**  **% (95 % CI)** | | **Phase 2+1 IgG,**  **% (95 % CI)** |
| **A. Seropositivity rates (exposure, past or recent/active infections)** | | | | | | | | | |
| South Reunion island (pregnant women), 2014 | | **1.3** | | | **0.3** | **20.1**  **(17.2 – 22.5)** | **1.3** | | **0.3** |
| Reunion island  (general population), 2009 | | - | | | **-** | 6.8  (6.7 – 6.9) | - | | - |
| Canada, 1996-1998 | | - | | | 2.2 | 4.3 | - | | 3,8 ; 4.4 |
| Netherlands, 2007-2008 | | - | | | - | - | 3.4 | | 1.15 |
| Netherlands, 2007-2009 | | 2.9 | | | **-** | 9.0 | 2.8 | | **-** |
| Antigua-Barbuda, 2009-2011  - | | | | -- | **-** | 5.3  ( -1.3 - 11.9) | - | | **-** |
| Jamaica, 2009-2011 | | - | | | **-** | 2.1  ( -2.0 - 6.2) | - | | **-** |
| Montserrat, 2009-2011 | | - | | | **-** | 6.6  ( -6.1 - 19.3) | - | | **-** |
| St. Kitt-Nevis, 2009-2011 | | - | | | **-** | 13.6  (3.5 - 24.7) | - | | **-** |
| All Caribbean islands, 2009-2011 | | | - | | **-** | 2.3  (0.9 - 3.7) | - | | **-** |
| **B. Seroprevalence rates (probable infections)** | | | | | | | | | |
| South Reunion island (pregnant women), 2014 | | **-** | | | **-** | **4.7**  **(3.4 – 5.9)** | **-** | **-** | |
| Reunion island  (general population), 2009 | | - | | | **-** | 3.9  (3.8 - 4.0) | - | - | |
| France, 1996 | | - | | | - | 0.15  (0.08 – 0.22) | - | **-** | |
| Canada, 1996-1998 | | - | | | 0.0 | 0.2 | - | 0.0 | |
| Denmark, 1996-2002 | | 0.8 | | | 0.7 | 19.7 | 0.7 | 8.5 | |
|  | | | | | | | | | |
